# Serum Proteomics of Older Patients Undergoing Major Cardiac Surgery: Identification of Biomarkers Associated With Postoperative Delirium

**DOI:** 10.3389/fnagi.2021.699763

**Published:** 2021-08-11

**Authors:** James Rhee, Alexandra Kuznetsov, Tina McKay, Margaret Lyons, Nicholas Houstis, Jennifer Mekkonen, Breanna Ethridge, Reine Ibala, Eunice Hahm, Jacob Gitlin, J. Sawalla Guseh, Robert Kitchen, Anthony Rosenzweig, Shahzad Shaefi, Adam Flaczyk, Jason Qu, Oluwaseun Akeju

**Affiliations:** ^1^Department of Anesthesia, Critical Care, and Pain Medicine, Massachusetts General Hospital, Harvard Medical School, Boston, MA, United States; ^2^Corrigan Minehan Heart Center, Massachusetts General Hospital, Harvard Medical School, Boston, MA, United States; ^3^Department of Anesthesia, Critical Care, and Pain Medicine, Beth Israel Deaconess Medical Center, Harvard Medical School, Boston, MA, United States

**Keywords:** postoperative delirium, proteomics, SOMAscan, TruCulture, IL-6, TIMP-1, PDE3A, cardiopulmonary bypass

## Abstract

**Background:**

Postoperative delirium (POD) is an acute altered mental state commonly encountered after cardiac surgery. The pathophysiological mechanisms underlying POD remain unclear. We aimed to identify circulating proteins significantly altered after major cardiac surgery with cardiopulmonary bypass (CPB). We also aimed to enable inferences on associations with POD.

**Methods:**

Serum and whole blood samples were collected before CPB (*n* = 16 patients; *n* = 8 with POD) and again from the same patients on postoperative day 1. All patients were clinically evaluated for POD on postoperative days 1–3. An aptamer-based proteomics platform (SOMAscan) was used to quantify serum protein abundance in patients with POD compared with non-POD controls. We also performed a lipopolysaccharide (LPS)-based *in vitro* functional analysis (TruCulture) on whole blood samples from patients with POD and non-POD controls to approximate surgical stress. Cytokine levels were determined using a Luminex immunoassay.

**Results:**

Cardiac surgery with CPB resulted in a significant (*p*_adj_ < 0.01) change in 48.8% (637 out of 1,305) of proteins detected by SOMAscan. Gene set enrichment showed that the most impacted biological processes involved myeloid cell activation. Specifically, activation and degranulation of neutrophils were the top five highest-scoring processes. Pathway analyses with the Kyoto Encyclopedia of Genes and Genomes (KEGG) showed that metabolic enzymes, particularly those of glycolysis, were elevated in serum concentration after surgery. Several proteins were significantly increased postoperatively in patients diagnosed with POD relative to the non-POD controls, with interleukin-6 (IL-6) showing the greatest fold-change. LPS stimulation of whole blood samples confirmed these findings. Linear regression analysis showed a highly significant correlation between Confusion Assessment Method (CAM) scores and CPB-mediated changes in cGMP-inhibited 3′,5′-cyclic phosphodiesterase A (PDE3A).

**Conclusions:**

Cardiac surgery with CPB resulted in inflammasome changes accompanied by unexpected increases in metabolic pathways. In exploratory analyses, we found that POD was associated with changes in the expression level of various proteins, most notably IL-6 and PDE3A. This study and ongoing protein biomarker studies will likely help quantify risk or confirm the diagnosis for POD and increase understanding of its pathophysiological mechanisms.

## Introduction

Cardiopulmonary bypass (CPB) was introduced in the 1950s to allow optimal operative conditions for major cardiac surgery (Castellheim et al., 2008). During CPB, venous blood is drained from the body, oxygenated, and then replaced into the arterial system using non-pulsatile flow. CPB and surgical injury are associated with the release of damage-associated molecular patterns that bind to evolutionary conserved families of receptors like Toll-like receptors expressed on various cell types (Sandler et al., 2018). This may subsequently trigger leukocyte migration, release of inflammatory mediators, and recruitment of immune cells. Margraf et al. (2020) recently reviewed this topic. The marked systemic inflammatory response associated with CBP (Gu et al., 1998; Matata et al., 2000; Castellheim et al., 2008; Serrano et al., 2010; Reed et al., 2020), likely influenced by genetic determinants (Lehmann et al., 2006), is associated with increased morbidity and mortality (Cleveland et al., 2001; Tomic et al., 2005).

Delirium, an acute brain dysfunction characterized by disturbances in attention, awareness, and cognition that are not fully explained by a preexisting neurocognitive disorder (American Psychiatric Association, 2013; Oh et al., 2020), is a morbidity commonly associated with older patients who undergo cardiac surgery with CBP (Rudolph et al., 2009; Brown et al., 2018). It is independently associated with increased mortality, prolonged hospitalization, and long-term cognitive deficits (Oh et al., 2020). An improved understanding of the systemic inflammatory response associated with CPB may elucidate putative mechanistic links with delirium. However, the expression pattern of a limited set of proteins is unlikely to lend principle insights into the large-scale complex interactions underlying inflammation associated with CBP. Thus, simultaneous quantification and analyses of a vast number of proteins that span logarithmic folds of abundance are necessary.

Therefore, we analyzed protein concentrations, both before and after CPB, of older patients undergoing major cardiac surgery [(*n* = 16 patients; *n* = 8 with postoperative delirium (POD)] using SOMAscan, a large-scale, multiplexed, and sensitive aptamer-based proteomics discovery tool. We found that cardiac surgery results in a significant change (*p*_adj_ < 0.01) in 48.8% of proteins (*n* = 1,305) analyzed. Changes in the inflammasome were accompanied by unexpected increases in metabolic pathways. In exploratory analyses, we found that POD was associated with changes in the expression level of various proteins, namely, interleukin-6 (IL-6), tissue inhibitor of metalloproteinases-1 (TIMP-1), and cGMP-inhibited 3′,5′-cyclic phosphodiesterase A (PDE3A), and that POD samples displayed an exaggerated response to lipopolysaccharide (LPS) stimulation.

## Materials and Methods

### Selection of Patients and Data Collection

This was a preliminary analysis of data obtained from patients enrolled in the Minimizing Intensive Care Unit Neurological Dysfunction with Dexmedetomidine-induced Sleep (MINDDS) trial. The details of the study about the MINDDS trial have previously been published (Shelton et al., 2018). Inclusion and exclusion criteria can be found at clinicaltrials.gov (NCT02856594). Patients underwent a baseline pre-randomization assessment for the inclusion and exclusion criteria of this study.

We screened for delirium during the pre-randomization assessment using the 3-min Confusion Assessment Method (CAM). Patients were screened for POD two times daily (before midday and past midday with at least 6 h between tests) beginning on postoperative day 1 using the long version of the CAM, until postoperative day 3. The CAM was administered by MINDDS research coordinators who obtained training and certification in delirium assessment *via* the Hospital Elder Life Program developed at the Beth Israel Deaconess Medical Center and now part of the American Geriatrics Society (AGS) CoCare portfolio and performed several initial assessments under the direct supervision of an experienced clinician or someone already certified. POD was also assessed with a structured chart review beginning on postoperative day 1 until postoperative day 3 by performing a text search for the diagnosis of “delirium” or “delirious” in the medical record.

### Blood Sample Collection and Handling (TruCulture and SOMAscan)

For SOMAscan, blood samples were collected before surgery and the morning of postoperative day 1 in a plastic serum separator tube (BD Vacutainer tube, BD Life Sciences, Franklin Lakes, NJ, United States) and allowed to clot for 30 min, followed by centrifugation at 383 × *g* at 22°C for 30 min. The supernatant was then collected, aliquoted, and stored at −20°C for 24 h, followed by transfer to −80°C until further processing. Samples were shipped on dry ice to SomaLogic (Boulder, CO, United States) for aptamer-based protein identification and quantification, as well as assay calibration and normalization.^1^ For *ex vivo* LPS stimulation, whole blood was collected in heparin tubes, with 1 ml of each sample added to pre-warmed TruCulture tubes (Myriad RBM, Austin, TX, United States) containing 2 ml of medium alone or medium with LPS (*Escherichia coli* O55:B5; 100 ng/ml). Samples were incubated at 37°C for 24 h for the stimulation reaction, after which liquid supernatants were aliquoted, frozen, and shipped on dry ice to Myriad RBM for analysis. Cytokine levels (ng/ml) were quantified by a 48-plex Luminex assay.

### Statistical Analyses

Significant differences in biomarker concentrations in the delirium cohort compared with the control group were assessed by using a repeated-measure ANOVA with the Šídák’s multiple comparisons test for each protein detected using GraphPad Prism (version 8.4.3) (GraphPad Software, San Diego, CA, United States). The Kolmogorov–Smirnov normality test was used to determine if the data were normally distributed. The Mann–Whitney *U* test was used for associations between continuous and categorical variables.

The relationship between preoperative to postoperative change in biomarker levels and CAM score was evaluated using linear regression, with significance determined from a two-tailed test of the beta coefficient. The nominal *p*-value from this significance test is reported along with the *R*^2^ goodness-of-fit metric. The *p*-value distribution of associations between change in protein level and CAM score was used to estimate false discovery rates. All calculations were performed in *R* with false discovery rates computed using the q-value package.

To characterize the biological processes most impacted by surgery with CPB, we performed differential gene expression analysis and gene set enrichment analysis (GSEA; Subramanian et al., 2005). For GSEA, proteins were ranked by decreasing test-statistic, and enrichment scores and *p*-values were calculated using the gseGO, gseKEGG, and gseMKEGG functions, in the clusterProfiler R package (version 3.18.1) (Yu et al., 2012) for the gene ontology (GO), KEGG, and modulated KEGG databases, respectively. The *p*-values for all enrichments were adjusted for multiple comparisons using the Benjamini–Hochberg method. Analysis was performed by using RStudio Version 1.3.2093.

## Results

### Changes During Cardiopulmonary Bypass Surgery

**FIGURE 1:**
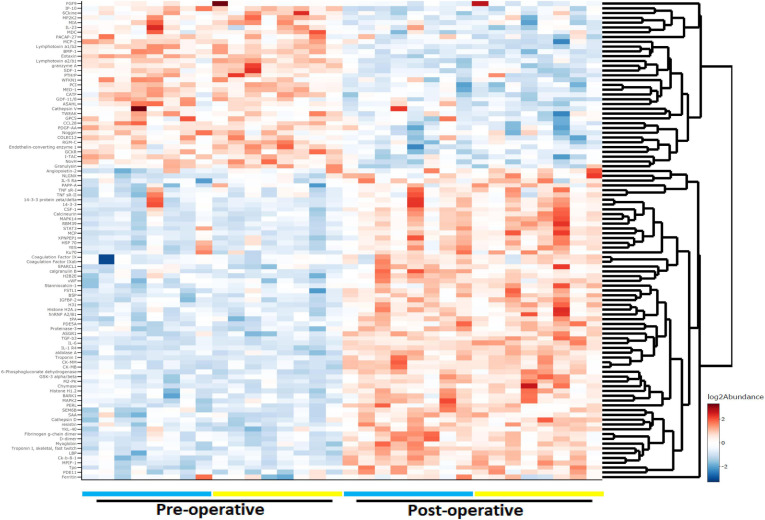
Heat map and dendrogram of preoperative and 24-h postoperative proteomes. The top 100 proteins with the most significant change (*p*_adj_ < 3e10^− 6^) at 24 h after surgery with cardiac bypass are listed. Patients with (*n* = 8) and without (*n* = 8) postoperative delirium (POD) are represented by yellow and blue bars, respectively.

We analyzed 1,305 proteins and found that the relative abundance of 828 (63.4%) proteins was altered significantly over the course of surgery with a *p*_adj_ < 0.05 (paired linear model) and 637 (48.8%) with a *p*_adj_ < 0.01. Figure 1 shows a heat map depicting the top 100 proteins whose levels differed most dramatically between the preoperative and postoperative states (*p*_adj_ < 3e10^–6^; Supplementary Dataset 1). Among the highest scoring proteins were known heart and skeletal muscle biomarkers, such as creatine kinase isoenzymes M and MB (CKM and CKMB), as would be expected after major cardiac surgery. Mediators of inflammation and tissue repair were highly upregulated after cardiac surgery, including cytokines and chemokines involved in both innate and adaptive immune responses. Specifically, significant increases were observed for IL-6 (*p*_adj_ = 7.66e10^–10^), granulysin (*p*_adj_ = 7.24e10^–9^), serum amyloid A-1 (*p*_adj_ = 1.52e10^–8^), macrophage colony-stimulating factor-1 (*p*_adj_ = 3.27e10^–8^), and interleukin-23 (*p*_adj_ = 3.50e10^–7^).

### Gene Set Enrichment and Pathway Analyses

SOMAscan detects proteins that span a wide range of biological functions, disease pathophysiology, and subcellular distribution. Cytokines constitute about 20% of the cognate proteins recognized by its aptamer library, with the remaining 80% being hormones (3%), protease inhibitors (5%), growth factors (13%), proteases (17%), kinases (20%), receptors (21%), and structural proteins (1%) [SomaLogic, (2014), *An Overview of SomaLogic and the SOMAscan Assay*, Company document]. While 47% of the proteins are classified as secretory, the rest of them are either intracellular (25%) or possess extracellular domains (28%). Given that approximately half of the 1,305 SOMAscan proteins were significantly changed by surgery with CPB and these proteins represent extensive biological diversity, it was important to determine which cellular functions were most impacted.

**FIGURE 2:**
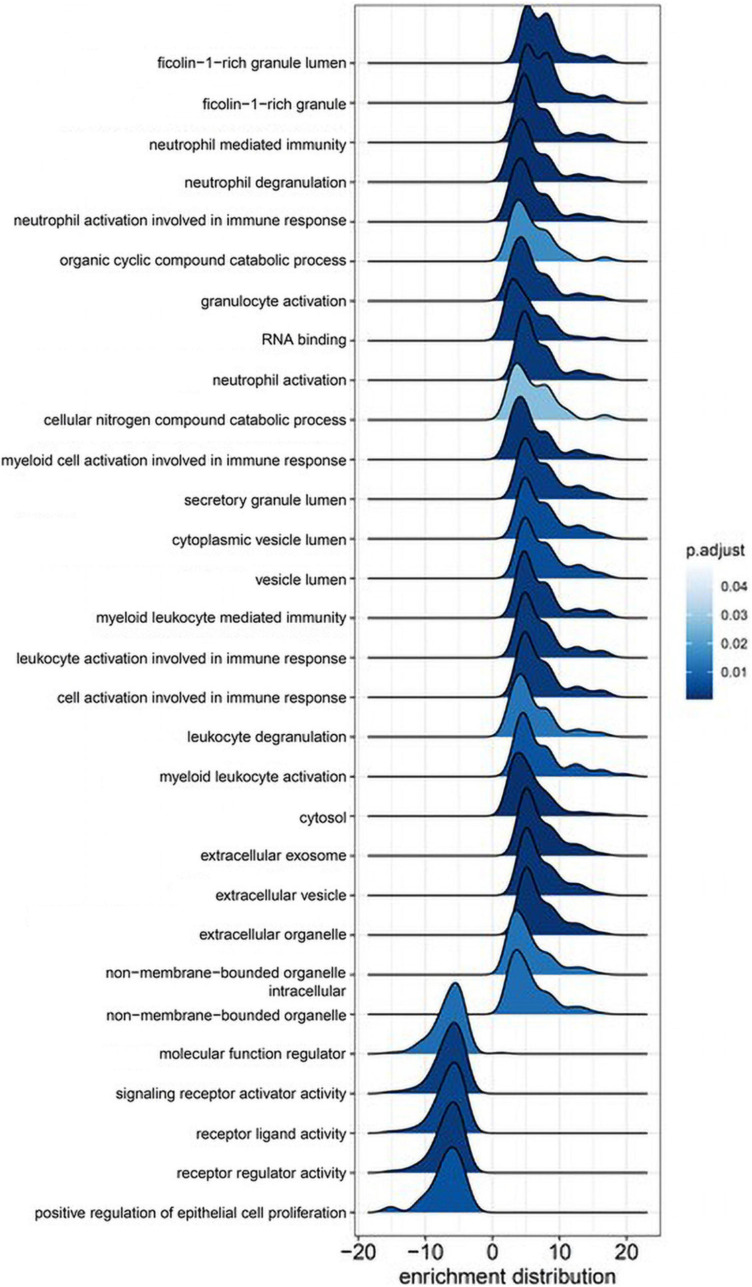
Gene ontology gene set enrichment analysis (GSEA). Immune cell activation and function are highly associated with proteomic changes during cardiac bypass surgery. Ridge plots depict the distribution of core enriched proteins, with positive enrichment scores representing increased expression after surgery.

Gene set enrichment analysis using GO curated sets was performed to identify biological processes affected by surgery with CPB (Figure 2). Leukocyte functions were highly represented, particularly those governing myeloid cell activation. The top five highest-scoring processes involved neutrophil migration and degranulation. The two most enriched functions were related to the protein ficolin-1, a key component of complement activation in the innate immune response that is expressed on granulocytes and monocytes (Holmskov et al., 2003).

**FIGURE 3:**
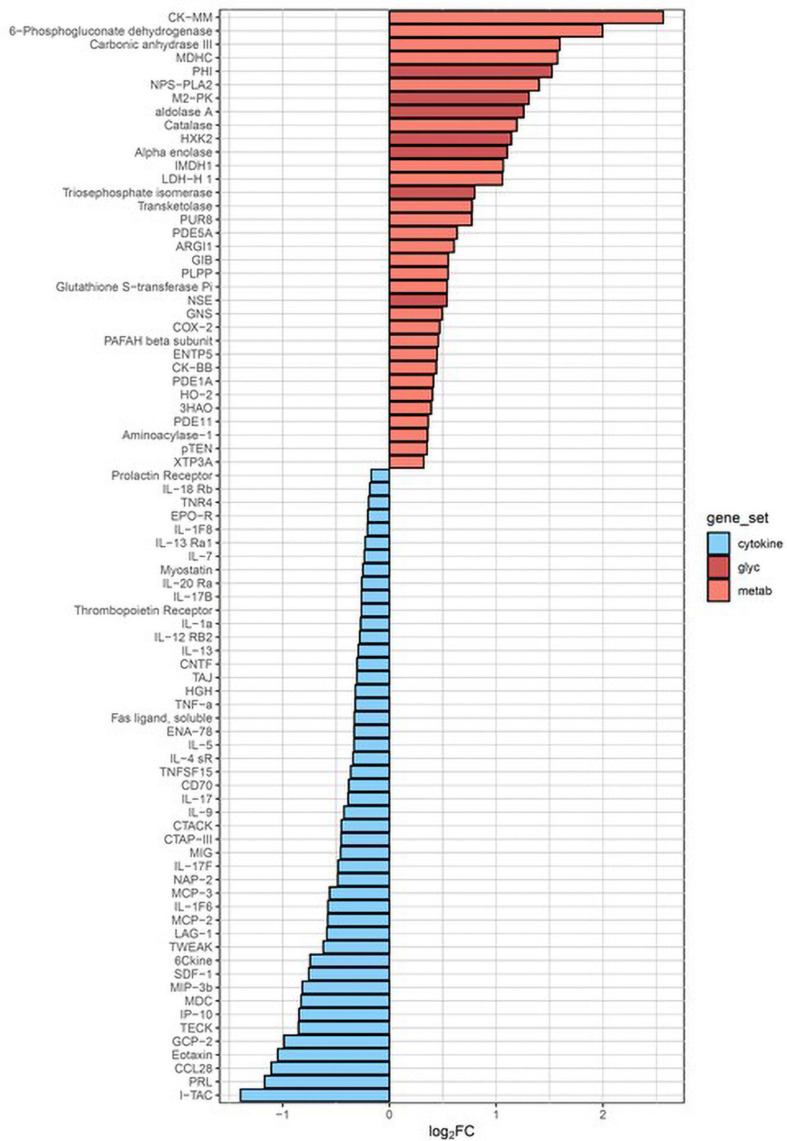
KEGG pathway analysis. The two biological pathways most altered by cardiopulmonary bypass surgery involve upregulation of metabolism (red bars) and downregulation of cytokine–receptor interactions (blue bars). The dark red bars all represent enzymes of glycolysis. PHI, glucose-6-phosphate isomerase; M2-PK, pyruvate kinase isozyme M2; HXK2, hexokinase 2; NSE, neuron-specific enolase.

KEGG pathway analysis (Figure 3) revealed two pathways altered post-CPB with *p*_adj_ < 0.3, metabolism (*p*_adj_ = 0.013), and cytokine–receptor interactions (*p*_adj_ = 0.096). The former was upregulated, and the latter, somewhat surprisingly, was downregulated. In particular, glycolysis was strongly impacted, with alpha-enolase, neuron-specific enolase, hexokinase 2, triosephosphate isomerase, glucose-6-phosphate isomerase, aldolase A, and pyruvate kinase M2, all significantly increased in serum concentration after surgery and depicted by the dark red bars. Certain chemokines with roles in homeostatic maintenance and restriction of leukocyte migration, such as CCL19 (MIP-3b), CCL25 (TECK), CCL27 (CTACK), CXCL11 (I-TAC), and CXCL12 (SDF-1) (Moser and Willimann, 2004; Mantovani et al., 2006; Shachar and Karin, 2013; Karin and Wildbaum, 2015), all had lower serum concentrations after CPB. Of the 47 proteins assigned to this KEGG pathway, CXCL11 had the greatest overall fold-decrease in the postsurgical state, with a *p*_adj_ = 1.34 × 10^–6^ (Supplementary Dataset 2).

### Delirium-Associated Proteomic Changes

**FIGURE 4:**
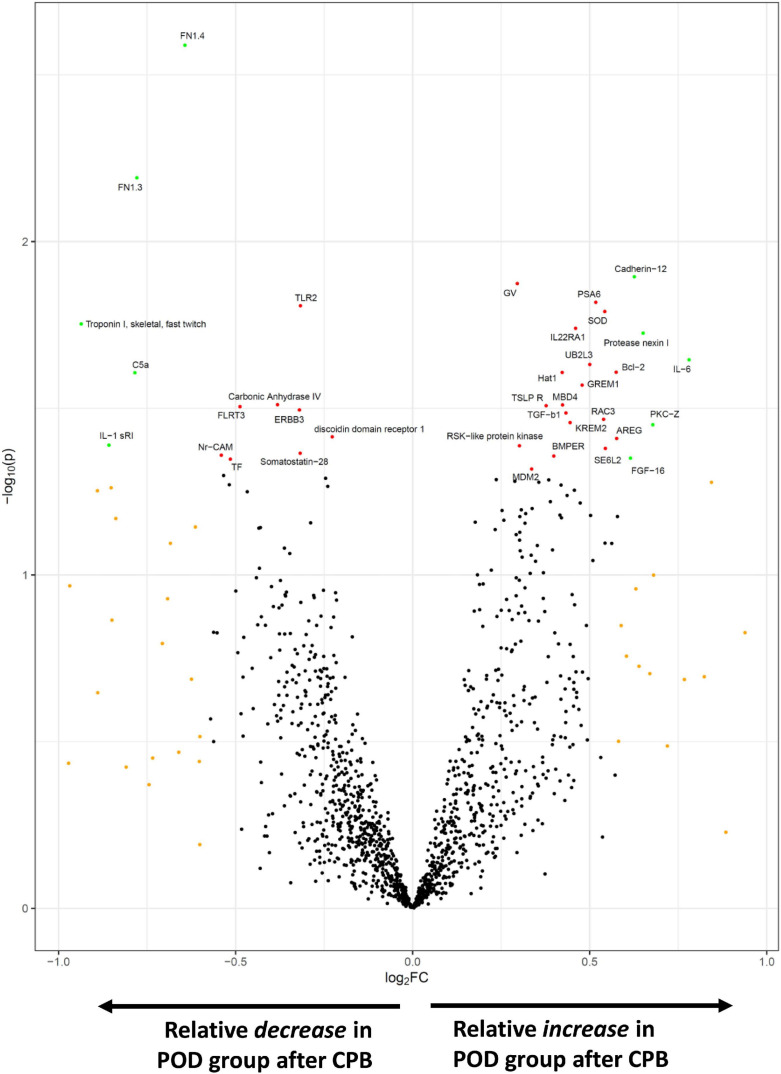
SOMAscan proteins associated with delirium. The volcano plot shows those proteins most differentially changed during surgery in the POD vs. non-POD groups. Fold change (FC) is calculated by (postoperative/preoperative level)_POD group_ ÷ (postoperative/preoperative level)_non–POD group_. Those proteins in orange satisfied absFC > 1.5, red achieved *p*-value < 0.05, and green met both *p*-value and FC criteria.

In exploratory analyses, we assessed for differences in protein biomarker levels between the POD and non-POD groups. There were no statistically significant group differences (*p* < 0.05) in key variables between POD and non-POD groups, including mean age, sex, BMI, and CPB time (Table 1). Importantly, levels of tissue injury in the two groups as assessed by circulating levels of heart and skeletal muscle biomarkers were similar (Supplementary Figure 1). We compared the change in each level of biomarker over the course of surgery between the POD and non-POD groups (Supplementary Dataset 3). Ten proteins (green dots, Figure 4) met threshold criteria for fold change (FC) > 1.5 (absolute log_2_FC > 0.59) and *p*-value < 0.05 (−log_10_*p*-value > 1.3). Of these proteins, IL-6 notably demonstrated the greatest relative increase in the POD group. The others included cadherin-12, protease nexin I, protein kinase C zeta, and fibroblast growth factor 16.

**TABLE 1:** Clinical features of the control and delirium cohorts for the SOMAscan analysis.

**Factors**	**Control (*n* = 8)**	**Delirium (*n* = 8)**	***p*-value**
Age (years)	74 ± 6.9	75 ± 6.5	0.82
Sex (female:male)	5:3	5:3	>0.9999
BMI (kg/m^2^)	28.18 ± 4.55	29.29 ± 5.02	0.88
Cardiopulmonary Bypass Time (min)	134.8 ± 58.7	149.5 ± 34.9	0.38
PROMIS Physical T-Score	44.23 ± 10.34	43.01 ± 8.94	0.70

*The data are shown as mean ± SD. Statistical significance determined using the Mann–Whitney *t*-test with *p* < 0.05 considered significant.*

**FIGURE 5:**
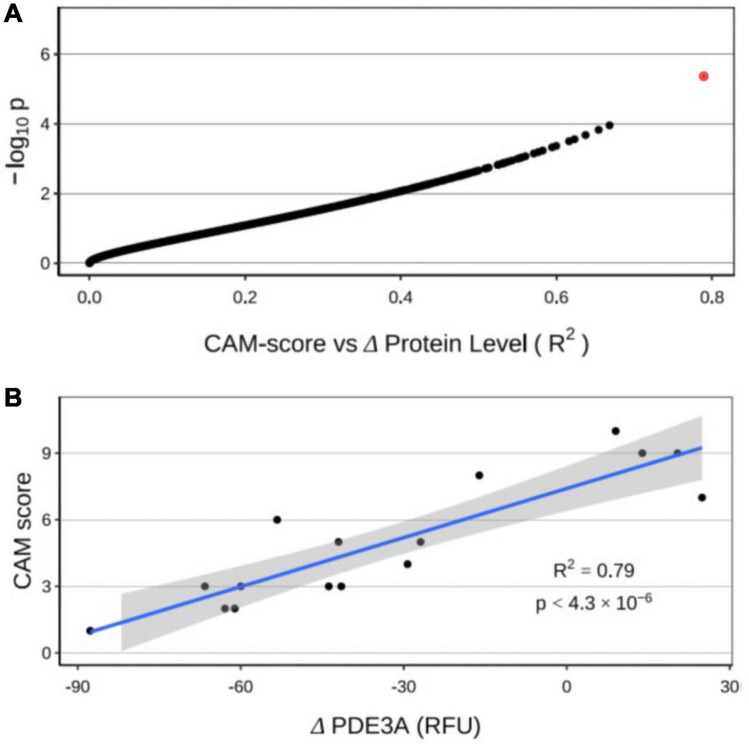
**(A)** Regression of CAM score against changes in protein levels from preoperative to postoperative states. The red dot represents cGMP-inhibited 3′,5′-cyclic phosphodiesterase A (PDE3A). **(B)** Significant correlation of individual changes in PDE3A concentration with CAM score.

Using CAM score as a quantitative phenotype rather than a binary one, we performed a regression of maximum CAM scores of individuals over the postoperative period against the change in protein level from preoperative to postoperative states (Figure 5A). Sixty proteins displayed *p*_adj_ < 0.05, with one having a *p*_adj_ < 0.01 (Supplementary Dataset 4). This protein, PDE3A, demonstrated the highest correlation between change over the course of CPB and CAM score (*R*-squared = 0.79). Figure 5B shows individual CAM scores plotted against the change in PDE3A. Patients with non-delirium (CAM < 5) showed the greatest relative decrease in PDE3A, while patients with delirium (CAM ≥ 5) had mild decrease or moderate increase in PDE3A.

### *In vitro* Stimulation of Whole Blood

Having identified a set of secreted factors whose levels post-CBP are more altered in the setting of POD, we addressed the possibility that circulating leukocytes in whole blood samples from POD and non-POD patients would respond differently to an inflammatory trigger *in vitro*. Blood was “activated” by LPS (TruCulture, Myriad RBM), an immune stimulus widely used to elicit inflammatory responses, to approximate surgical stress. A panel of 48 cytokines was quantified in each sample by Luminex immunoassay. We measured the baseline “control” level of each of these proteins preoperatively and postoperatively and then the “activated” level after LPS treatment.

To confirm that the immunoaffinity-based Luminex assay recapitulated the effect of surgery with CPB found in the aptamer-based SOMAscan, we first examined the overall cytokine expression in preoperative vs. postoperative samples. Approximately 31% (15 out of 48) of the proteins in our Luminex array were significantly changed (*p*_adj_ < 0.05) in the post-CPB samples (Supplementary Figure 2). We observed marked increases in several well-established inflammatory biomarkers and acute phase reactants, such as IL-6 (*p*_adj_ = 8.15e^–9^), C-reactive protein (CRP, *p*_adj_ = 2.31e^–8^), TIMP-1 (*p*_adj_ = 4.46e^–4^), von Willebrand factor (vWF, *p*_adj_ = 0.0053), ferritin (FRTN, *p*_adj_ = 0.011), and IL-8 (*p*_adj_ = 0.036). Supplementary Figure 3 depicts the difference in POD vs. non-POD groups in terms of how protein concentration changed with surgery. Similar to our SOMAscan results, IL-6 (*p*_adj_ = 0.023) again showed the most significant elevation, with a postoperative change over 2-fold more in the POD group than in the non-POD group.

**FIGURE 6:**
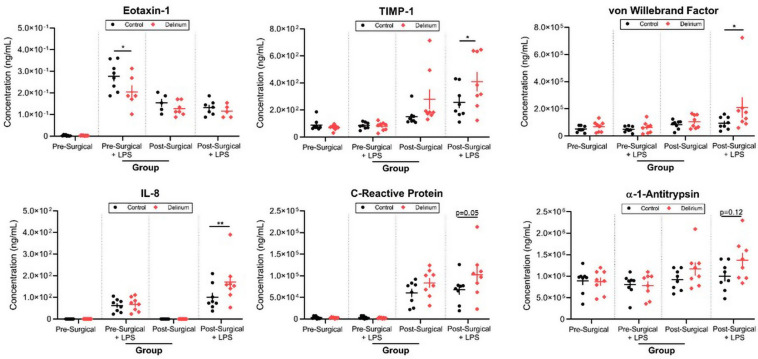
Lipopolysaccharide (LPS) stimulation of preoperative and postoperative whole blood. Samples from patients with delirium exhibited differential responses to LPS treatment as measured by several inflammatory markers. TIMP-1, tissue inhibitor of metalloproteinases 1; IL-8, interleukin 8; LoD, limit of detection. *p < 0.05 and **p < 0.01.

Finally, we analyzed the effect of LPS stimulation in preoperative and postoperative samples in order to unmask any potential difference in immune cell reactivity in patients with POD. LPS triggered an increase in nearly half of the inflammatory markers surveyed. There were significant increases in TIMP-1 (*p*_adj_ = 0.042), vWF (*p*_adj_ = 0.033), and IL-8 (*p*_adj_ = 0.0075) in postsurgical samples from POD patients treated with LPS (Figure 6). The increase in eotaxin-1 in response to LPS was significantly lower in presurgical samples from POD patients vs. presurgical samples from non-POD patients (*p*_adj_ = 0.012).

## Discussion

Proteomics offers a relatively quick, quantifiable method to survey biological fluids and tissue (Grandi et al., 2011; Khan et al., 2011; Baranyi and Rothenhausler, 2013; Westhoff et al., 2015; Fong et al., 2019b). Abundance, localization, and modification of signaling proteins may reflect upstream genomic and transcriptomic variation, which are often more challenging to broadly assess in a timely and cost-effective manner. In this study, we used SOMAscan (SomaLogic, Boulder, CO, United States), a highly multiplexed aptamer-based platform, which has previously been used to identify biomarkers in a variety of clinical cohorts (Sattlecker et al., 2014, 2016; Marion et al., 2016; Murota et al., 2016; Nishikawa et al., 2016; Petek et al., 2016; Fong et al., 2019a). We found that cardiac surgery with CPB resulted in the modulation of nearly half of a diverse subset of the human proteome. Our results were enriched for proteins implicated in neutrophil and monocyte activity, highlighting a putative role for innate immunity in the pathogenesis of POD. We noted that cardiac surgery with CPB has been shown to modulate leukocyte recruitment and transmigration (Rossaint et al., 2012), much more than cardiac surgery without CBP (Tomic et al., 2005). Specifically, our findings support a prominent role for ficolin-mediated activation of the lectin pathway of complement during cardiac surgery with CPB, likely in response to damage and pathogen-associated molecular patterns, and CPB circuitry (Garred et al., 2009, 2016; Michalski et al., 2019). In addition to regulating coagulation, ficolins augment inflammatory cytokine release and leukocyte chemotaxis.

Pathway analyses showed that surgery with CPB is associated with a significant upregulation in glycolytic enzymes that may support the high bioenergetic requirement of inflammation. It is tempting to speculate that such a rise in anaerobic respiration also reflects an attempt to compensate for an impairment in oxidative respiration. However, further characterization of mitochondrial protein regulation and function in the setting of CPB is warranted. This study also uncovered differences in molecular phenotypes between POD and non-POD groups after cardiac surgery. We found a disproportionate elevation in the cytokine IL-6 in patients with POD. This is consistent with the study by Vasunilashorn et al., who used mass spectrometry to identify proteins elevated in patients with delirium preoperatively, including CRP, and on postoperative day 2, namely IL-2 and IL-6 (Vasunilashorn et al., 2019). Furthermore, we found a highly significant correlation between CAM scores and PDE3A, with increasing POD severity associated with an inability to downregulate PDE3A. Phosphodiesterases degrade cAMP and cGMP, two critical second messengers involved in many physiological processes, including learning, memory, and cognition. Interestingly, inhibition of phosphodiesterases in neurologic diseases has been the focus of many preclinical and clinical studies (Wu et al., 2018; Bhat et al., 2020), with particular attention on PDE3 (Yanai and Endo, 2019).

Since there were no significant differences in baseline serum levels of cytokines preoperatively between POD and non-POD groups, we used an *in vitro* approach to uncover differential responses to an inflammatory stimulus. LPS activates both innate and adaptive immune responses akin to the tissue injury and bacteremia introduced by surgery. We postulated that LPS may unmask leukocytes primed for a heightened inflammatory response. Whole blood from our patients was assayed for their protein signature before and after exposure to LPS (TruCulture, Myriad RBM). In both SOMAscan and TruCulture approaches, TIMP-1 and IL-6 were higher in the POD group after surgery. LPS treatment of postoperative blood modulated the levels of many critical immune mediators and, in regard to IL-8 and TIMP-1, significantly more in the POD group. These findings supported the notion that there is a heightened level of inflammatory priming in whole blood leukocytes that may contribute to POD. vWF was significantly higher in samples from patients with POD treated with LPS, underscoring a possible hypercoagulable state in those more vulnerable to POD. It was also intriguing that there was relatively less eotaxin-1 in preoperative samples from POD patients after stimulation by LPS, as this chemokine has been associated with cognitive decline, aging, and Alzheimer’s disease (Huber et al., 2018). Interestingly, in certain contexts, such as traumatic brain injury, its level is inversely correlated with poor outcomes (Chaban et al., 2020).

We used two independent proteomic assays with completely different methods of detection to identify proteins that may be associated with POD. Significant findings were shared between these orthogonal methods, thus enhancing confidence that these proteins play important roles in delirium pathogenesis. The changes in a wide range of cytokines reported in this study reflect the systemic inflammation likely triggered by cardiac surgery with CBP. In turn, they may mediate the activation, propagation, or attempted resolution of the pathophysiological mechanisms that underlie POD. IL-6, in particular, has received considerable attention, as its increase may herald susceptibility to POD (Liu et al., 2013, 2018; Capri et al., 2014; Kline et al., 2016; Neerland et al., 2016). TIMPs and matrix metalloproteinases have been implicated with neurodegenerative diseases (Gardner and Ghorpade, 2003; Mroczko et al., 2013; Baranger et al., 2014). However, to our knowledge, TIMP-1 has not previously been associated with POD.

Our study has several important limitations. First, given that our sample size with POD was relatively modest, our findings were exploratory and hypotheses generating. Second, we obtained and analyzed serum samples at two discrete time points. Thus, our findings may not reflect rapid and shorter-lasting proteomic changes (less than 24 h) and do not uniformly coincide with the clinical onset of delirium. The changes we described in this study may represent both pathological mechanisms and compensatory efforts to mitigate the effects of surgery with CPB. Nevertheless, the proteomic signals we detected on postoperative day 1 may benefit prognostication prior to the diagnosis of POD. Future serial sample collection will help develop temporal patterns for each biomarker and allow more precise clinical correlation. Third, SOMAscan does not detect posttranslational modification of proteins, and in fact these modifications and non-specific interactions may affect the ability of the modified aptamers to recognize their cognate proteins. Fourth, our *in vitro* studies used a single dose of LPS, and future efforts may entail dose ranging of this and other immune stimuli. Finally, this was a substudy of the MINDDS trial where patients were randomized to placebo or dexmedetomidine intervention, to which we are still blinded in this ongoing clinical trial. Thus, the incidence of delirium or proteomic findings on postoperative day 1 may have been modified by dexmedetomidine. Therefore, this study, like all similar studies, needs replication in larger and different patient cohorts and should be further interpreted in the context of dexmedetomidine. Findings from future studies may also be correlated with clinical outcomes (e.g., length of hospital stay and mortality) previously associated with delirium.

Modulation of the immune response to cardiac surgery with CPB and its effect on the central nervous system may contribute to the development of POD. In ongoing and future studies, the tools described in this report may enable objective approaches to preemptively identify patients at risk for POD and predict its severity and those of associated clinical outcomes.

## Data Availability Statement

The raw data supporting the conclusions of this article will be made available by the authors, without undue reservation.

## Ethics Statement

The studies involving human participants were reviewed and approved by Partners Healthcare Institutional Review Board. The patients/participants provided their written informed consent to participate in this study.

## Author Contributions

OA designed the study. JR and OA drafted the manuscript. JM, BE, RI, EH, and JG recruited the participants in this study, collected their information, and processed blood samples. JR, AK, TM, ML, NH, RK, and JGu performed the data analyses. JR, AK, AR, SS, JQ, and OA made intellectual contributions and edited the manuscript. All authors contributed to the article and approved the submitted version.

## Conflict of Interest

The authors declare that the research was conducted in the absence of any commercial or financial relationships that could be construed as a potential conflict of interest.

## Publisher’s Note

All claims expressed in this article are solely those of the authors and do not necessarily represent those of their affiliated organizations, or those of the publisher, the editors and the reviewers. Any product that may be evaluated in this article, or claim that may be made by its manufacturer, is not guaranteed or endorsed by the publisher.

## Footnotes

1
http://somalogic.com/wp-content/uploads/2017/06/SSM-071-Rev-0-Technical-Note-SOMAscan-Data-Standardization.pdf

## Supplementary Material

Supplementary Figure 1 Cardiac and skeletal muscle markers in patients with delirium (D) and non-delirium (ND) presurgery and postsurgery with cardiopulmonary bypass (CPB). Plots depict minimum, first quartile, median, third quartile, and maximum values. CKM and CKMB, creatine kinase isoenzymes M and MB; PKM2, pyruvate kinase muscle isoenzyme M2.

Supplementary Figure 2 Heat map and dendrogram of preoperative and 24-h postoperative proteomes. Proteins in the TruCulture assay with the most significant change (*p*_adj_ < 0.05) at 24 h after surgery with cardiac bypass are listed. Patients with and without POD are represented by yellow and blue bars, respectively.

Supplementary Figure 3 TruCulture proteins associated with delirium. The plot shows those proteins most differentially changed during surgery in the delirium group vs. the non-delirium group. Fold change (FC) was calculated by (postoperative/preoperative level)_POD group_ ÷ (postoperative/preoperative level)_non–POD group_. Those proteins in orange satisfied absFC > 1.5 and green met both *p*_adj_ < 0.05 and FC criteria.
