# Supplementary figures and images for: Serum Proteomics of Older Patients Undergoing Major Cardiac Surgery: Identification of Biomarkers Associated With Postoperative Delirium
Source: Front Aging Neurosci. 2021 Aug 11;13:699763. doi: 10.3389/fnagi.2021.699763 (PMC8386117; doi:10.3389/fnagi.2021.699763)

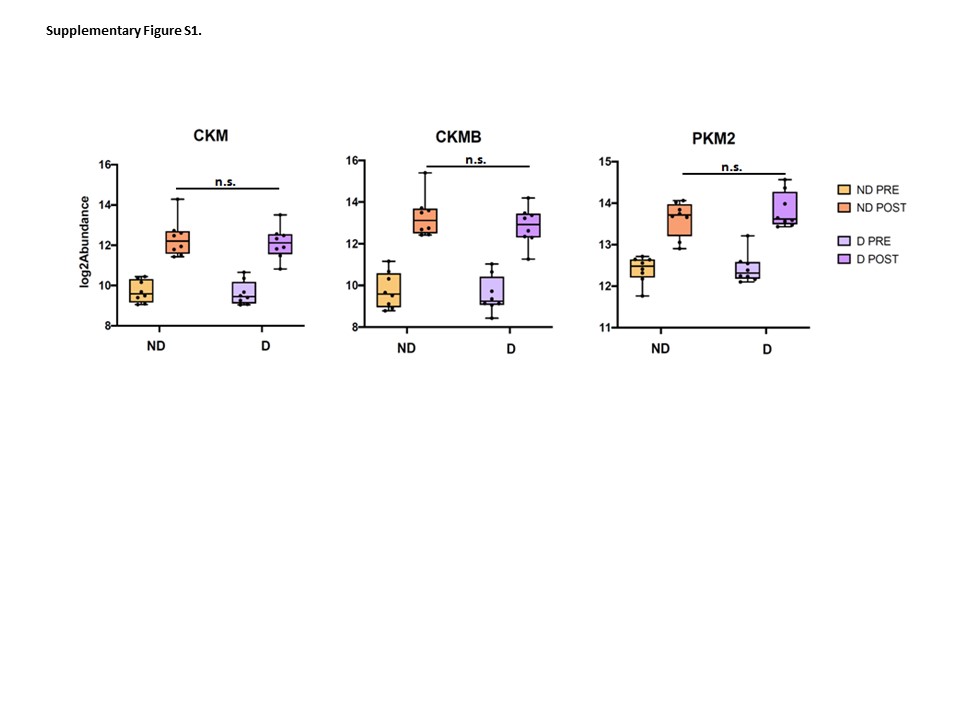

Supplement: Supplementary Figure 1 — Cardiac and skeletal muscle markers in patients with delirium (D) and non-delirium (ND) presurgery and postsurgery with cardiopulmonary bypass (CPB). Plots depict minimum, first quartile, median, third quartile, and maximum values. CKM and CKMB, creatine kinase isoenzymes M and MB; PKM2, pyruvate kinase muscle isoenzyme M2. [file Image_1.jpeg]

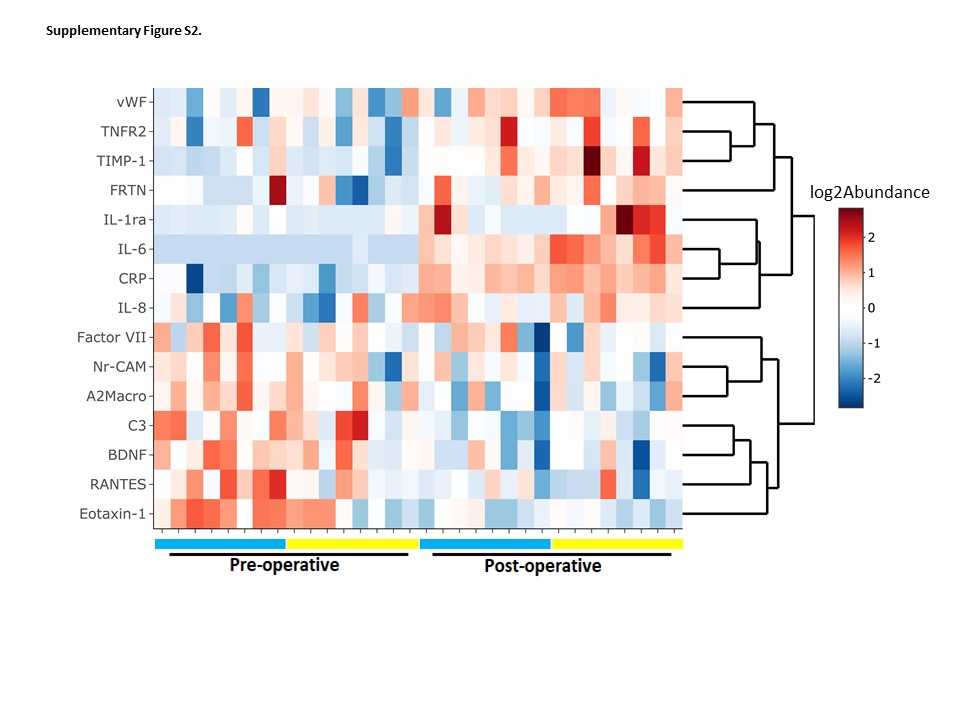

Supplement: Supplementary Figure 2 — Heat map and dendrogram of preoperative and 24-h postoperative proteomes. Proteins in the TruCulture assay with the most significant change (padj < 0.05) at 24 h after surgery with cardiac bypass are listed. Patients with and without POD are represented by yellow and blue bars, respectively. [file Image_2.jpeg]

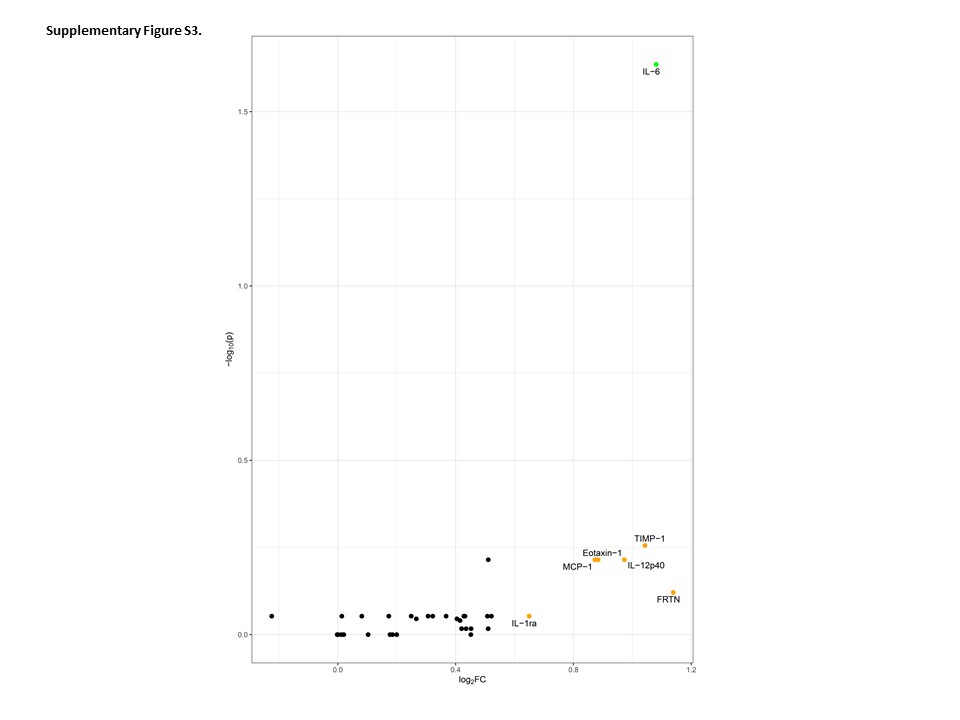

Supplement: Supplementary Figure 3 — TruCulture proteins associated with delirium. The plot shows those proteins most differentially changed during surgery in the delirium group vs. the non-delirium group. Fold change (FC) was calculated by (postoperative/preoperative level)POD group ÷ (postoperative/preoperative level)non–POD group. Those proteins in orange satisfied absFC > 1.5 and green met both padj < 0.05 and FC criteria. [file Image_3.jpeg]
